# Fatty Acid Composition of the Muscle Lipids of Five Fish Species in Işıklı and Karacaören Dam Lake, Turkey

**DOI:** 10.1155/2014/936091

**Published:** 2014-07-20

**Authors:** Ozcan Baris Citil, Leyla Kalyoncu, Oguzhan Kahraman

**Affiliations:** ^1^Department of Animal Nutrition and Nutritional Diseases, Faculty of Veterinary Medicine, University of Selcuk, 42075 Konya, Turkey; ^2^Department of Biology, Faculty of Science, University of Selcuk, 42075 Konya, Turkey

## Abstract

Total fatty acid composition of muscle lipids in some fish species (*Cyprinus carpio* (Işıklı Dam Lake), *Tinca tinca* (Işıklı Dam Lake), *Scardinius erythrophthalmus* (Işıklı Dam Lake), *Cyprinus carpio* (Karacaören Dam Lake), and *Carassius carassius* (Karacaören Dam Lake)) was determined by gas chromatography. Polyunsaturated fatty acids (PUFAs) of *Cyprinus carpio* (Işıklı Dam Lake) were found higher than PUFA of other species. Palmitic acid was the highest saturated fatty acid (SFA) in *Tinca tinca* (24.64%). Oleic acid was the highest monounsaturated fatty acid (MUFAs) in *Cyprinus carpio* (Işıklı Dam Lake) (19.25%). The most abundant polyunsaturated fatty acid in *Scardinius erythrophthalmus* was docosahexaenoic acid (DHA) (17.94%). Total *ω*3 fatty acid composition was higher than the total *ω*6 fatty acids of *Cyprinus carpio* in both dam lakes. *ω*3/*ω*6 rates in *Cyprinus carpio* (Işıklı Dam Lake), *Tinca tinca, Scardinius erythrophthalmus, Cyprinus carpio* (Karacaören), and *Carassius carassius* were 2.12, 1.19, 2.15, 2.87, and 2.82, respectively.

## 1. Introduction

The composition of fat acids of sea and freshwater fish has distinct differences [1]. The difference of fat acids compositions of fats in seafood depends on some factors. Those factors can be considered as diet, geographic conditions, ambient temperature, and seasonal changes, according to the season of hunting, body length, sex, species, and fat content [2].

The lipid and the type of fatty acids vary mainly according to species, seasons (especially the breeding season), the feeding areas, food structure, water temperature, and pollution, whether species culture or wild forms, diet, geographic conditions, ambient temperature, and seasonal changes, according to the season of hunting, body length, sex, fat contents, and the body portions [3, 4].

It has demonstrated that the consumption of food containing long-chain fatty acids undertakes role in reducing the risk of coronary heart disease, in treating hypertension and cardiac arrhythmia, in preventing sudden death, in reducing the incidence of diabetes, in the improvement of skin diseases, in reducing symptoms of rheumatoid arthritis, in preventing impaired vision, alzay, and schizophrenia, and in reducing problems such as hyperactivity in slowing disease, inhibitors to fight cancer. As a result, deficient nutrition of *ω*3 has been identified as indeficient development vision and nerve tissue in babies [5].

Eicosapentaenoic acid (EPA) and DHA in fish are also important physiologically. For development of sea fish's brain and retina, those essential oils are also necessary [6]. After identification of therapeutic aspects of EPA and DHA, it has been understood that they protect the body against especially migraine headaches, heart attacks, depression, rheumatic fever, certain forms of cancer, diabetes seen in adults, high cholesterol, high blood pressure, cardiovascular diseases, and some allergies. As a result of this, the pills produced from fish oil are commercially marketed [7].

No reports have yet been published about the fatty acid composition of* Cyprinus carpio* (Işıklı Dam Lake),* Tinca tinca* (Işıklı Dam Lake),* Scardinius erythrophthalmus* (Işıklı Dam Lake),* Cyprinus carpio* (Karacaören Dam Lake), and* Carassius carassius* (Karacaören Dam Lake).* Cyprinus carpio*'s fatty acid composition varies in the reservoir. The aim of this study is to characterize and compare these species in terms of their fatty acid composition. In addition, another aim of study is to determine the differences between fatty acid compositions of* Cyprinus carpio*'s and other species.

## 2. Materials and Methods

### 2.1. Study Area

Işıklı and Karacaören Dam Lakes are freshwater lakes in Isparta region of Turkey. It is a large lake in Isparta and situated in southwestern part of Turkey. It is located at around 37°N 30°E and has an area of 25–80 km². Karacaören and Light Isparta and Burdur Lake Dam are located within the city borders. It is about 270 meters above sea level having average depth of 80 meters. The highest water level is 275.5 meters with catchment area of 5582 km^2^. The elevation from the sea level of Karacaören Lake Dam is about 2000 meters. Dam construction started in 1972 and was completed in 1989. Lake irrigates 9,500 hectares in downstream and serves purposes such as electricity generation and fishing [8]. Işıklı and Karacaören Dam Lake is also an important wetland for birds.* Cyprinus carpio* (Işıklı Dam Lake),* Tinca tinca* (Işıklı Dam Lake),* Scardinius erythrophthalmus* (Işıklı Dam Lake),* Cyprinus carpio* (Karacaören Dam Lake), and* Carassius carassius* (Karacaören Dam Lake) are the most common fish species in such lakes.

### 2.2. Sample Collection

*Cyprinus carpio* (Işıklı Dam Lake),* Tinca tinca* (Işıklı Dam Lake),* Scardinius erythrophthalmus* (Işıklı Dam Lake),* Cyprinus carpio* (Karacaören Dam Lake), and* Carassius carassius* (Karacaören Dam Lake) were used in this study. They were obtained from local fishermen in both dam Lakes in July. The season chosen for analysis was summer (hot season). Nine individuals were sampled from all species for total lipid extraction and fatty acid analyses in this season. All fish had almost the same size (average weight 1.000 kg) and age (over 2-year-old). Fish were transported in ice to the laboratory. Dorsal muscle tissues of fish were taken as samples. The samples were frozen at −24°C until analyses. At the beginning of analysis, the samples were allowed to equilibrate at room temperature.

### 2.3. Fatty Acid Analysis

Total lipids of fish were extracted with chloroform: methanol (2 : 1 v/v) according to Folch et al. [9]. The fatty acids in the total lipid were esterified into methyl esters by saponification with 0.5 N methanolic NaOH and transesterified with 14% BF_3_ (v/v) in methanol [10].

Fatty acid methyl esters (FAMEs) were prepared from nine samples obtained from the 5 fish species in July. The FAMEs were analyzed on a HP (Hewlett Packard) Agilent 6890N model gas chromatograph (GC), equipped with a flame ionization detector (FID) and fitted with a DB-23 capillary column (60 m, 0.25 mm i.d, and 0.25 *μ*m). Injector and detector temperatures were 270 and 280°C, respectively. Column temperature program was 190°C for 35 min then increased at 30°C/min up to 220°C where it was maintained for 5 min. Total run time was 36 min. The used carrier gas was helium (1 mL/min). GC analysis of FAMEs was performed with 3 replications.

The fatty acids were identified by comparison of retention times with known external standard mixtures (Alltech), quantified by a Shimadzu Class-VP software, and the results were expressed as percentage distribution of fatty acid methyl esters. C4:0 Alltech part number: 622040, C20:3 Alltech part noumber: 62302030, C20:5 Alltech part number: 623205, C22:2 Alltech part number: 6232202, C4:0 Alltech part number: 622040, C23:0 Alltech part number: 623230 of single fatty acid standards were used. Alltech part numbers: 625002, 625003, 625005, 625009, 625010, 625023, and 625029 mix fatty acids standards were used.

### 2.4. Statistical Analysis

Nine items of fish samples were analysed in July and each sample in triplicate. The average results of peak area were offered as means ± SD. FID responses were corrected to weight percent automatically. The results were submitted to analysis of variance (ANOVA), at 0.05 significance level, using SPSS 19.0. The mean values were compared by Tukey's test.

## 3. Result and Discussion

The fatty acid composition in muscles of* Cyprinus carpio* (Işıklı Dam Lake),* Tinca tinca* (Işıklı Dam Lake),* Scardinius erythrophthalmus* (Işıklı Dam Lake),* Cyprinus carpio* (Karacaören Dam Lake), and* Carassius carassius* (Karacaören Dam Lake) are presented in Table 1. Thirty-two fatty acids were identified and evaluated in the fish samples.

DHA was the major polyunsaturated fatty acid in* S*.* lucioperca* (22.48%) and* T. capoeta* (17.65%). This fatty acid was determined as the predominant fatty acid in* S. lucioperca* [11]. Concerning* Cyprinus carpio* (Işıklı Dam Lake),* Tinca tinca* (Işıklı Dam Lake),* Scardinius erythrophthalmus* (Işıklı Dam Lake), and* Carassius carassius* (Karacaören Dam Lake), oleic acid was the major fatty acid. Similarly, oleic acid was identified as the major fatty acid in* Cyprinus carpio*, in Apa Dam Lake, Turkey [12], and in* P. anatolicus* in Sugla Lake, Turkey [13]. In the present study, palmitic acid was the most abundant fatty acid in* Tinca tinca*.

In this study, the major SFA was palmitic acid and other predominant SFAs were stearic and myristic acid in all fish species. These results were similar to those reported by Görgün and Akpinar [14], for* Alburnus chalcoides,* and Karaçali et al. [15], for* Cyprinus carpio*. In the present study, total SFAs in Tinca* tinca* (35.34%) were higher than those of* S. erythrophthalmus* (33.52%),* C. carpio *(Işıklı) (30.55%),* Carassius carassius* (26.41%), and* C. carpio *(Karacaören) (24.95%). Total SFA was determined as 19.94% in* C. regium* obtained from the Tigris river [16], 31.49% in* C. capoeta* [17], and in* V. vimba tenella* [18].

The predominant MUFA was oleic acid and it was followed by palmitoleic acid in all the species except* Cyprinus carpio*. Similar results were observed by Cengiz et al. [16], for Liza abu, Kalyoncu et al. [18], for* V. vimba*, Ateş et al. [19], for* Salmo trutta* macrostigma, and Guler et al. [11], for* S. lucioperca*. Higher levels of palmitoleic acid have been described as a characteristic of* C. carpio* [12]. In our study, the oleic acid in* C. carpio *(19.25%) was significantly higher than in the other species. Total MUFA in* C. carpio* (38.17%) was also significantly higher than in the other species. The high level of oleic acids was found by comparison to* Scardinius erythrophthalmus* (Işıklı Dam Lake),* Carassius carassius* (Karacaören Dam Lake),* Tinca tinca* (Işıklı Dam Lake), and* Cyprinus carpio* (Karacaören Dam Lake). Total MUFA was reported as 36.10–37.15% in* C. carpio* (Ozparlak, 2013) [12], 33.01–39.34% in* Salmo turutta* by Ateş et al. [19] 45.67–50.17% in mirror carp in Orenler Dam Lake by Karaçali et al. [15], 40.4–45.3% in* V. vimba tenella* by Kalyoncu et al. [18], and 21.12–40.04% in* Alburnus chalcoides* [12].

In the present study,* C. carpio* was rich in PUFA, especially DHA, arachidonic acid, EPA, docosapentaenoic acid (DPA), and linoleic acid. These results matched with Guler et al. [20], who reported that EPA, DHA, and arachidonic acid are the most abundant PUFA in* C. carpio* muscle lipids in Beyşehir Lake, Turkey. In all species except* C. carpio* (Karacaören), DHA was identified as the major PUFA. Similarly, DHA was the major PUFA in* C. carpio* [12]. In the present study, DHA in* S. erythrophthalmus* (17.94%) was significantly higher than in* T. tinca* (17.93%),* C. carpio* (Işıklı) (17.71%),* C. carassius* (14.17%) and* C. carpio* (13.18%).* C. carpio* was rich in PUFA in winter (55.38%), especially DHA (16.61%), and eicosapentaenoic acid (15.60%) which are higher than that reported by Guler et al. [21] in* C. carpio*. In the present study, EPA was determined as the predominant PUFA in* C. carpio* (Karacaören). Similar result was obtained by Guler et al. [20] for* C. carpio* in spring. Total PUFA in* C. gibelio* (43.90%),* P. anatolicus* (50.96),* S. lucioperca* (50.64),* T. tinca* (37.59),* V. vimba tenella* (20.66), and* C. capoeta* (51.86). Total PUFA was 31.76% in* Alburnus mossulensis* Cengiz et al. [16], 45.95% in* C. gibelio* Ozparlak, 2013 [12], and 25.6–32.7% in* V. vimba tenella*, seasonally [18]. In the present study, total PUFA was higher than total SFA and total MUFA in* C. carpio*. These results matched with Guler et al. [11] who reported that PUFA is higher than total SFA and total MUFA in* S. lucioperca*. Kminkova et al. [22] also reported that* C. carpio* from Benesow Lisno Fishery, Czech Republic, had high contents of PUFA compared to SFA and MUFA.

EPA and DHA have beneficial properties for the prevention of human coronary artery disease [6]. In our study, among the *ω*3 series,* C. carpio* was a good source of EPA and DHA. EPA + DHA were 18.27, 18.12, 18.28, 34.09, and 24.41% in* Cyprinus carpio* (Işıklı Dam Lake),* Tinca tinca* (Işıklı Dam Lake),* Scardinius erythrophthalmus* (Işıklı Dam Lake),* Cyprinus carpio* (Karacaören Dam Lake), and* Carassius carassius* (Karacaören Dam Lake), respectively. EPA + DHA were higher than that reported by Guler et al. (2007) [11] in* S. lucioperca* Beysehir Lake in autumn and spring (28.27–29.23%), in Turkey. Similarly, Kalyoncu et al. [18] determined that Egirdir Lake fish is a good source for *ω*3 fatty acids, particularly EPA and DHA, and should be recommended for dietary inclusion to reduce the risks of cardiovascular disease.

## 4. Conclusion

In the current study, fatty acid compositions of five important fish species in Işıklı and Karacaören Lake were researched. The reservoirs of* C. carpio*'s fatty acid composition obtained from Işıklı and Karacaören was different from each other.* C. carpio* (Karacaören) had more *ω*3 fatty acids, important for human health, than other fish species. The percentages of total *ω*3 fatty acids were higher than those of the total *ω*6 fatty acids in all species.* C. carpio* may be a good dietary source of *ω*3 PUFA.

## Figures and Tables

**Table 1:** Fatty acid composition of fish grown in Karacaören and Işıklı Dam Lake.

Fatty acids	*Cyprinus carpio *	*Tinca tinca *	*Scardinius erythrophthalmus *	*Cyprinus Carpio *	*Carassius carassius *
	(Işıklı)	(Işıklı)	(Işıklı)	(Karacaören)	(Karacaören)
C 4:0	0.11 ± 0.01^c^	0.00 ± 0.00^d^	0.54 ± 0.03^a^	0.21 ± 0.02^b^	0.00 ± 0.00^d^
C 10:0	0.02 ± 0.01^b^	0.00 ± 0.00^b^	0.40 ± 0.02^a^	0.00 ± 0.00^b^	0.00 ± 0.00^b^
C 12:0	0.14 ± 0.00^b^	0.06 ± 0.00^c^	0.46 ± 0.02^a^	0.05 ± 0.01^c^	0.11 ± 0.00^b^
C 13:0	0.10 ± 0.05^b^	0.11 ± 0.04^b^	0.59 ± 0.01^a^	0.00 ± 0.00^b^	0.00 ± 0.00^b^
C 14:0	3.03 ± 0.18^a^	1.41 ± 0.09^c^	3.16 ± 0.12^a^	2.29 ± 0.04^b^	2.43 ± 0.06^b^
C 15:0	0.17 ± 0.00^b^	1.02 ± 0.03^a^	0.27 ± 0.02^b^	0.31 ± 0.02^b^	1.01 ± 0.03^a^
C 16:0	19.57 ± 3.27^b^	24.64 ± 3.82^a^	19.21 ± 3.36^b^	16.65 ± 2.67^b^	16.04 ± 2.42^b^
C 17:0	1.46 ± 0.03^b^	0.26 ± 0.01^c^	2.28 ± 0.06^a^	1.78 ± 0.05^b^	1.97 ± 0.06^b^
C 18:0	3.28 ± 0.03^a^	3.35 ± 0.12^a^	3.70 ± 0.07^a^	2.23 ± 0.02^b^	2.64 ± 0.04^b^
C 20:0	0.92 ± 0.01^a^	0.96 ± 0.01^a^	1.14 ± 0.01^a^	0.92 ± 0.00^a^	0.92 ± 0.00^a^
C 21:0	0.76 ± 0.01^a^	0.25 ± 0.00^b^	0.94 ± 0.00^a^	0.24 ± 0.00^b^	0.63 ± 0.11^ab^
C 22:0	0.36 ± 0.01^a^	0.21 ± 0.00^b^	0.36 ± 0.01^a^	0.12 ± 0.11^b^	0.30 ± 0.03^a^
C 23:0	0.33 ± 0.09^a^	0.09 ± 0.00^b^	0.22 ± 0.13^a^	0.17 ± 0.14^a^	0.27 ± 0.01^a^
C 24:0	0.25 ± 0.15^b^	2.97 ± 0.86^a^	0.27 ± 0.00^b^	0.00 ± 0.01^b^	0.09 ± 0.08^b^
∑ SFA	**30.55**	**35.34**	**33.52**	**24.95**	**26.41**
C 14:1 *ω*5	1.07 ± 0.11^a^	0.85 ± 0.00^a^	1.10 ± 0.01^a^	0.40 ± 0.03^b^	0.87 ± 0.21^a^
C 16:1 *ω*7	11.79 ± 1.79^a^	10.20 ± 1.64^a^	11.60 ± 1.47^a^	7.43 ± 1.03^b^	9.71 ± 1.25^a^
C 18:1 trans	0.49 ± 0.21^a^	0.11 ± 0.00^b^	0.77 ± 0.03^a^	0.56 ± 0.09^a^	0.32 ± 0.11^a^
C 18:1 cis	19.25 ± 4.21^a^	10.47 ± 2.08^b^	16.07 ± 3.57^a^	5.80 ± 1.15^c^	15.23 ± 2.74^a^
C 20:1 *ω*9	4.61 ± 1.62^a^	5.56 ± 1.36^a^	4.80 ± 1.78^a^	5.74 ± 1.69^a^	5.82 ± 1.07^a^
C 22:1 *ω*9	0.69 ± 0.01^c^	2.67 ± 0.16^b^	0.46 ± 0.00^c^	6.27 ± 0.74^a^	5.68 ± 0.96^a^
C 24:1 *ω*9	0.27 ± 0.01^a^	0.06 ± 0.00^a^	0.18 ± 0.00^a^	0.18 ± 0.00^a^	0.19 ± 0.00^a^
∑ MUFA	**38.17**	**29.92**	**34.98**	**26.08**	**37.82**
C 18:2 trans	1.46 ± 0.26^a^	0.96 ± 0.31^a^	1.60 ± 0.23^a^	0.85 ± 0.14^b^	1.00 ± 0.12^b^
C 18:2 cis	6.47 ± 1.11^b^	13.06 ± 2.78^a^	4.78 ± 0.92^b^	7.14 ± 1.34^a^	5.20 ± 0.87^b^
C 18:3 *ω*6	0.27 ± 0.17^a^	0.07 ± 0.02^b^	0.19 ± 0.01^a^	0.18 ± 0.01^a^	0.39 ± 0.08^a^
C 18:3 *ω*3	0.95 ± 0.55^a^	0.29 ± 0.00^b^	0.85 ± 0.42^a^	0.40 ± 0.37^ab^	0.88 ± 0.24^a^
C 20:2 *ω*6	0.69 ± 0.02^b^	0.11 ± 0.01^e^	0.64 ± 0.01^c^	0.34 ± 0.01^d^	0.91 ± 0.24^a^
C 20:3 *ω*6	0.04 ± 0.00^b^	0.01 ± 0.00^c^	0.26 ± 0.00^a^	0.01 ± 0.00^c^	0.03 ± 0.00^b^
C 20:3 *ω*3	1.93 ± 0.37^a^	0.17 ± 0.13^e^	0.85 ± 0.02^d^	0.89 ± 0.01^c^	1.03 ± 0.86^b^
C 20:4 *ω*6	0.74 ± 0.04^a^	0.28 ± 0.05^d^	0.75 ± 0.17^a^	0.35 ± 0.01^c^	0.49 ± 0.02^b^
C 22:2 *ω*6	0.31 ± 0.03^e^	1.17 ± 0.11^d^	2.51 ± 0.26^b^	3.47 ± 0.42^a^	1.42 ± 0.13^c^
C 20:5 *ω*3	0.56 ± 0.00^c^	0.19 ± 0.00^c^	0.34 ± 0.00^c^	20.91 ± 5.74^a^	10.24 ± 3.35^b^
C 22:6 *ω*3	17.71 ± 5.04^a^	17.93 ± 5.41^a^	17.94 ± 5.75^a^	13.18 ± 3.68^b^	14.17 ± 4.37^b^
*∑* PUFA	**31.12**	**34.26**	**30.73**	**47.72**	**35.76**
*ω*3	21.15	18.58	19.99	35.38	26.32
*ω*6	9.97	15.68	10.74	12.34	9.43
*ω*3/*ω*6	2.12	1.19	2.15	2.87	2.82
Unknown	0.16 ± 0.02^d^	0.48 ± 0.08^c^	0.77 ± 0.01^b^	1.25 ± 0.04^a^	0.01 ± 0.00^e^

The highest value in the same line denoted with a, the lowest value was denoted with e and the values between a and e were denoted with others. If the value had been denotation of ab, bc, cd, de (or ed) it was found in the range of a and b, b and c, c and d, d and e (or e and d). a, b, c, d, e values for each sample were denoted with different letters in the same fraction. These values were significantly different at *P* < 0.05.
